# Activation of Erk in the anterior cingulate cortex during the induction and expression of chronic pain

**DOI:** 10.1186/1744-8069-4-28

**Published:** 2008-07-23

**Authors:** Feng Wei, Min Zhuo

**Affiliations:** 1 Department of Biomedical Sciences, University of Maryland Dental School, Baltimore, Maryland 20201, USA; 2 Department of Physiology, University of Toronto, Medical Sciences Bldg., Rm 3342, 1 King's College Circle, Toronto, Ontario, M5S 1A8, Canada

## Abstract

The extracellular signal-regulated kinase (Erk) activity contributes to synaptic plasticity, a key mechanism for learning, memory and chronic pain. Although the anterior cingulate cortex (ACC) has been reported as an important cortical region for neuronal mechanisms underlying the induction and expression of chronic pain, it has yet to be investigated whether or not Erk activity in the ACC may be affected by peripheral injury or in chronic pain state. In the present study, we use adult rat animal models of inflammatory and neuropathic pain and demonstrate that Erk signaling pathway in the ACC is potently activated after peripheral tissue or nerve injury. Furthermore, we demonstrate that mechanical allodynia significantly activated Erk activity at synaptic sites at two weeks after the injury. We propose a synaptic model for explaining the roles of Erk activity during different phases of chronic pain. Our findings suggest that cortical activation of Erk may contribute to both induction and expression of chronic pain.

## Short report

Recent studies have consistently indicated that Erk signaling cascade plays an important role in activity-dependent plasticity in the central nervous system (CNS) and may contribute to the molecular mechanisms underlying learning, memory and persistent pain. Previous studies have found that tissue and nerve injury transiently activates Erk pathway in the spinal dorsal horn neurons, and the activation of Erk is required for the central sensitization during the development of hyperalgesia and allodynia [1-5]. In supraspinal structures, it has been reported that activation of Erk in the amygdala is induced by peripheral injury [6], and the increased Erk activity in this region is acquired for behavioral sensitization to mechanical stimulation after injury [7]. The ACC has been found to be an important site for cortical regulation of nociception and persistent pain after amputation [8-11]. Long-term potentiation (LTP) in ACC neurons is the likely synaptic model for persistent pain [12-16]. Recent studies using pharmacological inhibitors has showed that the activity of Erk contributes to synaptic potentiation caused by LTP induction protocols [17], and that such inhibitors are relatively selective and do not affect basic synaptic transmission. However, little is known about the possible involvement of Erk in the ACC after tissue or nerve injury in adult animals.

**Figure 1:**
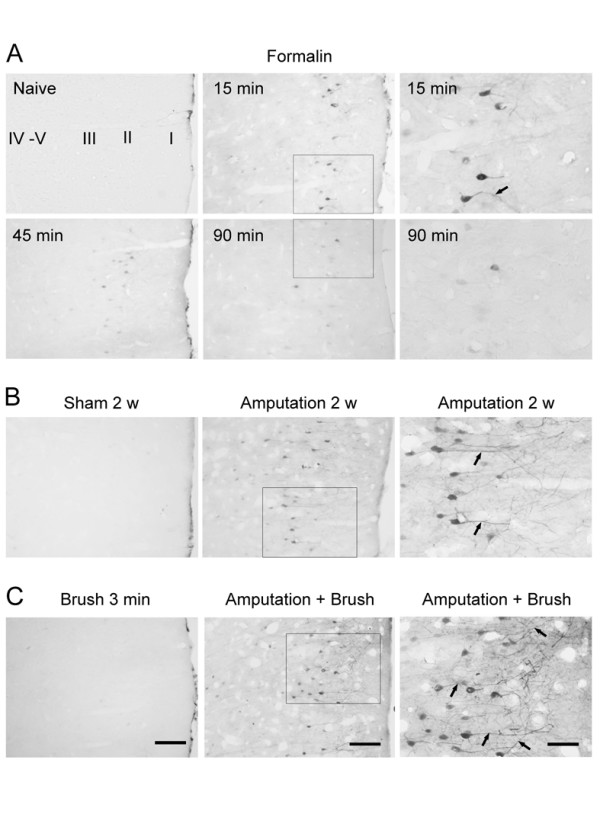
**Enhanced Erk activation in the ACC after tissue and nerve injury**. **A**. Immunohistochemical staining for phosphorylation of Erk illustrated time course-dependent activation of Erk in layer II neurons of the contralateral ACC after unilateral hindpaw injection of formalin (5%, 50 μl, n = 4–5 rats for each time point). **B**. The P-Erk expression in the layer II ACC neurons and their main apical dendrites (arrows) was increased at 2 weeks after the amputation of the unilateral hindpaw third digit (n = 5), compared to sham animals (n = 3). **C**. Mechanical stimulation by brushing hindpaw of digit amputation induced P-Erk expression in more number of layer II ACC neurons and the more distinctive apical dendrites at 2 weeks after the amputation (n = 5), compared to that in rats with amputation alone. There was not P-Erk activation in the ACC in normal animals after the brushing (n = 3). Left and middle columns: low power of the coronal ACC sections. Scale bar= 50 μm; Right column: enlarged layer II regions corresponding to the small rectangle areas in the middle column, respectively. Scale bar = 25 μm.

To determine the possible activation of Erk in the ACC during acute or chronic pain after peripheral injury, we carried out experiments in adult male rats using two different injury models. Activation of Erk was monitored by immunostaining with an antibody that detects the activated form of Erk (phosphorylated on both thr-202 and Try-204, P-Erk). For the first pain model, 5% formalin was injected into the dorsal part of the unilateral hindpaw as previously reported [18]. At three different time points between 15 min to 90 min after the formalin injection, rats were killed by rapidly anesthetized and perfused through the heart with fixative. We found that formalin injection induced a rapid increase in P-Erk expression in some of layer II neurons in the bilateral ACC at 15 min after tissue injury (Fig. 1A). The expression of P-Erk was mainly restricted in the cell bodies but weak in their dendrites (Fig. 1A). The activated Erk level in the ACC was reduced at 45 min and declined at 90 min after formalin injection. There was no obvious Erk activation in the deep layer neurons in the ACC. Thus, the P-Erk expression pattern in the region is different from that of immediate early genes, such as c-Fos, which is widely expressed in ACC neurons located in all layers after formalin injection as previously reported [18]. These findings suggest that Erk activation caused by tissue injury is likely occurring at subpopulation of the layer II neurons. It has been known that neurons in the Layer II/III receive ascending noxious inputs from the thalamus and communicate with other cortical areas [19]. Thus, the Erk activation in ACC neurons may play a role in Erk-dependent neuronal plasticity in the ACC during the induction or development of inflammatory pain.

Phantom pain is chronic pain occurring after losing or amputation of a part of limb or organ. Although the animal model for studying central mechanisms of phantom pain is rare, investigations of changes in central synapses in the ACC after amputation in animals may reveal molecular mechanism for plastic changes that are caused by amputation. We have previously demonstrated that amputation of a single digit resulted in a loss of synaptic cortical depression [20,9,11] in brain slice preparations, and caused LTP of synaptic responses in the ACC to peripheral sensory stimulation or local synaptic stimulation [11,21], demonstrating that central plasticity takes place in the synapses of ACC neurons after the amputation. Because the Erk activity is shown to be required for cingulate LTP [17], we decided to test if tissue and nerve injury after the amputation would activate the Erk pathway in the ACC neurons. As shown in Fig. 1B, we found that larger number of the layer II pyramidal-like neurons in the ACC expressed P-Erk immunoreactivity at two weeks after single digit amputation. Activation of Erk activity in the ACC is bilateral. Interestingly, the P-Erk expression in these neurons was present in cytoplasm of both cell body and dendrites (Fig. 1B). There was some P-Erk in neurons of the deeper layers, suggesting the possible interaction between neurons in different layers of the ACC. This data further indicates that there is a prolonged activation of Erk in the layer II neurons after amputation.

In addition to spontaneous pain and hyperalgesia, allodynia is the most common feature of pathological pain and is a painful response to a usually innocuous stimulus. Touch-evoked allodynia occurs often in patients with phantom pain after amputation. Most of previous studies focus on the activation of Erk at early time points after the injury, there is few studies for the possible involvement of Erk activity during allodynic stimulation. Therefore, we applied non-noxious mechanical stimuli (brushing) on amputated hindpaw in rats to identify if Erk signaling in the ACC may be activated. To our surprise, there was significantly enhanced activation of Erk in the ACC neurons after brushing the amputated hindpaw. In addition to the increased number of P-Erk labeled neurons in the layer II, we also observed that there was stronger expression of P-Erk in the dendrites distributed in the layer I (Fig. 1C), as compared with that in rats with amputation alone. Most of P-Erk labeled pyramidal neurons in the layer II exhibited strong immunoreactivity in the main and distal apical dendrites, which branched upward to the superficial layer I of the ACC (see Fig. 1C). We also observed that there were many P-Erk labeled granular-like neurons in the deeper layers of the ACC. Again, we found similar activation pattern at bilateral sides of the ACC. By contrast, non-noxious brushing alone in normal rats did not cause any detectable Erk activation in the ACC. The enhanced expression of P-Erk in ACC neurons and their dendrites after brushing normal skin of amputated hindpaw suggests that Erk activity are recruited at distal synapses in ACC neurons. It may contribute to local synaptic plasticity and/or neuronal modulation during allodynia after amputation.

**Figure 2:**
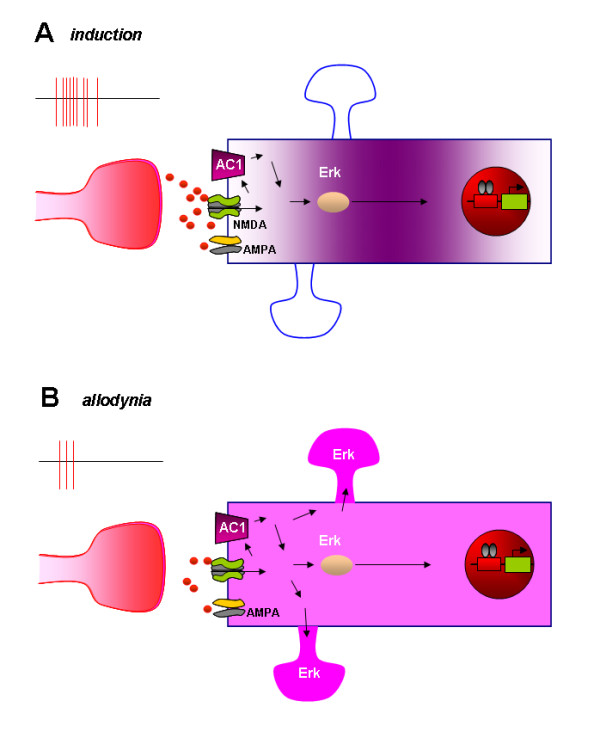
**A model for activation of Erk in the induction and expression phases of chronic pain**. **A**. In the induction phase of chronic pain, peripheral injury triggers glutamate release in the ACC synapses. Activation of NMDA receptors leads to an increase in postsynaptic Ca^2+^, and Ca^2+ ^binds to CaM and leads to activation of Ca^2+^-stimulated AC1. cAMP, a key second messenger, leads to activation of Erk, and PKA-dependent CREB. New protein synthesis is likely triggered as a consequence of CREB activation. **B**. In the expression phase of chronic pain (allodynia), a non-noxious stimuli triggers glutamate release in sensitized ACC synapses. Due to synaptic enhancement caused during the induction phase [13], glutamate triggers greater postsynaptic activation because AMPA and NMDA receptor mediated responses are likely enhanced after the injury [13,23]. Such postsynaptic sensitization makes activation of Erk at distal synaptic sites possible. Activated Erk at synaptic sites may contribute to AMPA receptor modulation, ion channel modulation and other synaptic modifications.

Our results provide the first evidence for the activation of Erk activity in the ACC neurons after tissue or nerve injury. More importantly, we show that the enhanced activation of Erk activity in synaptic sites in the layer II/III neurons during the touch-evoked allodynia after the amputation; suggesting the likely contribution of Erk activity to the central mechanisms underlying pathological pain. Different activation pattern of Erk in the inflammatory and amputation pain model suggest that Erk may contribute to different types of chronic pain in different manners. This is further supported by our observation of distal dendrite Erk activation during mechanical allodynia. Fig. 2 is a proposed synaptic model showing different subcellular location of P-Erk during two different phases of chronic pain: induction and expression of mechanical allodynia. The expression of p-Erk in the cytoplasm near the nucleus during the induction of chronic pain may play roles in the activation of a series of plasticity-related immediate early genes such as cAMP response element binding protein (CREB) [16]. In this case, P-Erk may trigger a series of plasticity-related signaling molecules that are important for inducing plastic changes in the ACC. In the expression phase of chronic pain (or mechanical allodynia here), in part due to prolonged plastic changes triggered in the induction phase, a normally non-noxious stimulus (i.e., non-noxious brushing of the skin) triggers a wide spread activation of Erk in ACC neurons. In addition to the cytoplasm, activation of Erk at synaptic sites is found. These activated Erk may contribute to rapid synaptic potentiation [17], the regulation of neuronal excitability [5], and other local modulations of synaptic transmission and neuronal excitability (see Fig. 2).

Although we cannot distinguish the contribution of Erk activities to pain perception, pain-related emotional responses or pain-related memory in the present study, we believe that such increased Erk activity may contribute to long-term plastic changes in the ACC caused by the injury or amputation. In fact, in brain slices, we demonstrate that Erk activity is required for synaptic potentiation in the ACC excitatory synapses [17]. One major function of Erk may contribute to cortical plasticity during the expression of behavioral allodynia, and serve as one of key signaling protein kinase in chronic pain [16].

## Materials and methods

### Animals

Adult male Sprague Dawley rats weighing 250–300 gram were used. Animals were kept in cages (2 animals per cage) at an ambient temperature of 20–25°C under a 12 hr light/dark cycle and had free access to food and water. We adhered to the ethical guidelines for investigation of experimental pain in conscious animals [22].

### Animal models of chronic pain

For inflammatory pain model, fifty microliters of a 5% formalin solution (dissolved in saline) were injected into the plantar surface of the left hindpaw. For rat model of amputation, under brief anesthesia with halothane, the third digit of the left hindpaw was amputated. In some cases, at 2 week after the amputation, a brush stimulus (with number 4 camel's hair artist's brush) was applied for 3 min by stroking vertically at the dorsal part of amputated hindpaw. At 15 min, 45 min, 90 min and 120 min after formalin injection, 2 week after the amputation, or 15 min after brush stimulus, rats were deeply anesthetized with halothane and perfused transcardially with 100 ml of saline, followed by 500 ml of cold 0.1 M phosphate buffer containing 4% paraformaldehyde. The brain was removed, post-fixed for 4 hr, and then cryoprotected. Coronal sections (25 μm) through the ACC were cut using a cryostat. Sham groups without formalin injection or amputation were performed as controls. Sections from sham and experimental animals were processed simultaneously for immunostaining.

### Immunohistochemistry

Immunostaining was performed using free-floating sections [4]. Briefly, the ACC sections were first treated with 0.75% Triton X-100 and 1% H_2_O_2 _in PBS for 1 hr, and then processed for 30 min in 3% normal goat serum, followed by incubation with anti-phospho-p44/42 Erk (Thr202/Tyr204) monoclonal antibody (diluted 1:500; Cell Signaling, Beverly, MA) overnight at room temperature. Secondary reactions with biotinylated goat anti-mouse immunoglobulin (1:400; Vector Laboratories, Burlingame, CA) for 1 h were followed by avidin-biotin-peroxidase complexes (1:100; Vector Laboratories) for 1 h. A nickel-intensified diaminobenzidine with glucose oxidase was used as the final chromogen. Sections were washed several times, mounted on gelatinized slides, dehydrated through a series of ethanol solutions, cleared in xylene, and covered with glass coverslips. Controls, performed by replacing primary antibody with 1% NGS in the protocol, exhibited no staining.

## Authors' contributions

The authors declare that they have no competing interests. FW and MZ participated in the conception, design, and interpretation of the study. FW carried out the experiments, FW and MZ wrote the manuscript.
